# Clinical Features and Cytokine Profile in Myositis Patients with Anti-EJ Autoantibodies Detected by a Novel Immunoprecipitation Assay

**DOI:** 10.1155/2019/1856180

**Published:** 2019-03-25

**Authors:** Yang Yang, Yangtengyu Liu, Li Huang, Li Wang, Ke Liu, Meidong Liu, Hui Luo, Xiaoxia Zuo, Yisha Li, Huali Zhang

**Affiliations:** ^1^Department of Rheumatology, Xiangya Hospital, Central South University, Changsha, Hunan, China; ^2^Department of Pathophysiology, Xiangya School of Medicine, Central South University, Changsha, Hunan, China; ^3^Sepsis Translational Medicine Key Laboratory of Hunan, Central South University, Changsha, Hunan, China

## Abstract

**Objective:**

This study aimed to clarify the clinical features, the serum level of autoantibodies, and cytokine of myositis patients with anti-EJ antibody, which targets glycyl tRNA-synthetase (GlyRS).

**Methods:**

Sera of 236 Chinese patients with myositis were screened for anti-EJ by a novel immunoprecipitation assay of flag-tagged GlyRS. Anti-EJ positive patients are evaluated for the clinical features and cytokine profile.

**Results:**

The sera from 4 of 236 adult myositis patients were found to carry the anti-EJ using established novel immunoprecipitation assay and immunoblotting. The prevalence of anti-EJ in our cohorts is about 1.7%. The decline of anti-EJ level was detected in two patients during disease remission. Interstitial lung disease and muscle weakness, but not skin involvement, are common clinical features of anti-EJ positive patients. Moreover, using a cytokine profile analyses, we found that the serum levels of IP-10, IL-6, MCP-1, and VEGF were significantly elevated in patients with anti-EJ and gradually decreased during disease remission of two patients, whereas IL-8 level was obviously reduced in these patients.

**Conclusion:**

The novel immunoprecipitation assay is suitable to detect and monitor the levels of anti-EJ autoantibody. The serum levels of anti-EJ, IP-10, IL-6, MCP-1, and VEGF may be related to disease activity in myositis patients with anti-EJ antibodies.

## 1. Introduction

Antisynthetase syndrome (ASS) has been characterized as the clinical combination of myositis, interstitial lung disease (ILD), fever, arthritis, Raynaud's phenomenon (RP), and Mechanic's hands with the presence of anti-aminoacyl-tRNA-synthetase antibodies (anti-ARS) [1]. ARS, which exist in cytoplasm ubiquitously, are a group of enzymes that catalyze the binding of amino acids to the cognate transfer RNA during translation process [2]. To date, there are eight distinct ARS autoantibodies that have been detected in 35-40% of idiopathic inflammatory myopathies (IIM) or myositis [3–6]. Anti-Jo1 (anti-histidyl-tRNA synthetase-Ab) are most commonly identified in 15-30% of IIM and in up to 90% of those patients with ILD [3, 7]. Autoantibodies targeting other ARS are less common (less than 5% prevalence per each) [1]. These antibodies have been discovered by the technique of immunoprecipitation using silver staining or radioisotope analyses to detect RNAs [8]. Currently, some anti-ARS including anti-alanyl (PL12), anti-threonyl (PL7), anti-isoleucyl (OJ), and anti-glycyl (EJ) tRNA-synthetase antibodies are also routinely diagnosed by immunoblot [9, 10].

Although all these anti-ARS antibodies represent the hallmark of the antisynthetase syndrome, several reports have attempted to associate anti-ARS antibodies specifically with distinct clinical features and prognosis [9, 11–13]. For example, it has been reported that ILD was more frequent whereas myositis was less common in patients with anti-PL7 or anti-PL12 compared to anti-Jo1 and patient survival was significantly lower in patients with anti-PL7/12 rather than anti-Jo1 [13]. A recent meta-analysis also revealed that patients with non-anti-Jo1 ARS had greater odds of fever and ILD compared to those with anti-Jo1 autoantibodies and the frequencies of myositis and arthralgia were almost 50% higher in patients with anti-Jo1 compared to non-anti-Jo1 ARS autoantibodies [14].

In the present report, we established a novel immunoprecipitation (IP) assay of HEK293 cell lysate overexpressing flag-tagged glycyl tRNA-synthetase (GlyRS) to screen anti-EJ antibody in sera from 236 myositis patients and confirmed the presence of anti-EJ antibody by an immunoblot. We sought to characterize the clinical significance of anti-EJ antibodies in this population and analyze the level of anti-EJ during disease remission and also performed the cytokines profile analysis in anti-EJ antibody positive sera using Bio-Plex Pro Human Cytokine 27-plex Assay.

## 2. Materials and Methods

### 2.1. Patients and Sera

Serum samples were obtained from 236 Chinese patients with myositis who had visited the Division of Rheumatology in Xiangya Hospital of Central South University (Changsha, Hunan, China) from 2012 to 2016 and stored at -80°C until analysis. PM and DM were defined by fulfillment of the Bohan and Peter criteria [15]. Severity of muscle weakness was classified into Grade 0 to Grade V according the criteria by experienced physician. Electromyogram was performed in these patients. Interstitial lung disease (ILD) was diagnosed based on the respiratory symptoms such as dyspnea and the presence of typical features including ground-glass opacities, reticulation, or honeycombing on high-resolution computed tomography (HRCT) chest scan, performed by an experienced radiologist. When available, forced vital capacity and lung carbon monoxide transfer factor were used to evaluate the pulmonary function. Serum samples from 20 healthy controls were also obtained. Our study was approved by the institutional review board at Xiangya hospital of Central South University (Approval number: 201703567). All study participants signed a written informed consent prior to participation in the study.

### 2.2. Serological Data

Antinuclear antibodies (ANA) were detected by indirect immunofluorescence (IIF) using HEp-2 cells with starting dilution of 1:160 in all myositis patient and HEp-2 is not a laryngeal cell line but is contaminated by HeLa (http://iclac.org/databases/cross-contaminations/). Antibodies against nRNP/Sm, Sm, SSA, Ro-52, SSB, Scl-70, JO-1, CENP B, nucleosome, histone, and ribosomal P-protein were detected using commercial line blot (EUROIMMUNE, Lubeck, Germany). The commercial myositis profile EUROLINE (DL1530-1601G, DL1530-1601-3G, DL1530-1601-4G) is not available in our clinical laboratory. The reference serum (YWN) in the present study has been detected positive for anti-EJ autoantibodies using commercial myositis profile EUROLINE (DL1530-1601-4G) at EUROIMMUN CN. Inc. in Hangzhou City.

### 2.3. Unlabeled Protein Immunoprecipitation Using Overexpressing Flag-Tagged GlyRS

We established a protein IP assay to detect anti-EJ in patients with myositis. Human HEK293 cells (ATCC CRL-1573) were grown in DMEM supplemented with 10% fetal bovine serum (Gibco), 100 U/ml penicillin, and 100 *μ*g/ml streptomycin (Invitrogen) in a humidified atmosphere of 5% CO_2_ at 37°C. Transient transfections were carried out using Megatran 2.0 (TT200003, Origene, USA) with pENTER-flag- GlyRS (glycyl tRNA synthetase) plasmid (CH810182, Vigene Biosciences, USA). After 48 hrs, cells were lysed in RIPA buffer (50 mM Tris–HCl, pH 7.4, 150 mM NaCl, 1% NP-40, 1% sodium deoxycholate, and 0.1% SDS) for western blot or protein immunoprecipitation. IP was performed according to protocols with minor modifications [16, 17]. Immunoprecipitated antigens were solubilized in 1× SDS-PAGE loading buffer and separated by 10% SDS-PAGE and transferred to PVDF membrane. The membrane was then detected with anti-flag antibodies (1:1000, F1804, Sigma). The secondary antibody used was HRP-conjugated anti-mouse IgG (1:10000, GAM007, Multi Science).

### 2.4. Immunoblotting of HEK293 Lysate Overexpressing Flag-Tagged GlyRS with Anti-EJ positive Serum

Human HEK293 cells overexpressing Flag-tagged GlyRS were lysed in RIPA buffer and separated by 10% SDS-PAGE and transferred to PVDF membrane. We tested the membrane in immunoblotting with an anti-EJ positive serum as reference serum diluted in 1:1000. The secondary antibody was HRP-conjugated anti-human IgG (1:5000, ab6759, Abcam).

### 2.5. Conventional Protein Immunoprecipitation

Human K-562 cell (ATCC CCL-243) were grown in RPMI-1640 supplemented with 10% fetal bovine serum (Gibco), 100 U/ml penicillin, 100 *μ*g/ml streptomycin (Invitrogen) in a humidified atmosphere of 5% CO_2_ at 37°C. Cells were freshly lysed in RIPA buffer for protein IP. IP assays from K-562 lysates using patient sera were performed in accordance with the following protocols. Briefly, 30 *μ*l of patient serum was incubated with 50 *μ*l of a 50% slurry of Protein G Magnetic Beads (Merck Millipore, Billerica, MA, USA) suspended in 5% bovine serum albumin (Sigma-Aldrich, St. Louis, MO, USA). Incubation was carried out for 1 h at room temperature (RT) with mixing on a rotary mixer. Beads were washed three times with 0.1% Tween 20 in PBS. Beads were then incubated with 200 *μ*g K-562 cell lysates overnight at 4°C under mild spinning conditions. After washing five times with 0.1% Tween 20 in PBS, immunoprecipitated protein was solubilized in 1× SDS loading buffer, separated by 10% SDS-PAGE, and transferred to PVDF membrane. Then, the membrane was detected with anti-GlyRS antibodies (1:1000, ab89522, Abcam). The secondary antibody used was HRP-conjugated anti-mouse IgG (1:5000).

### 2.6. Multiplex Cytokine Assays

Diluted serum specimens (1:4) were prepared for cytokine profile analysis in a 96-well plate utilizing the Bio-Plex Pro Human Cytokine 27-plex Assay (Bio-Rad, Hercules, CA). The 27-plex assay kit contains beads conjugated with monoclonal antibodies specific for interleukin (IL)-1RA, IL-1*β*, IL-2, IL-4, IL-5, IL-6, IL-7, IL-8, IL-9, IL-10, IL-12p70, IL-13, IL-17A, IL-15, Eotaxin, IFN-*γ*, IP-10, MCP-1, MIP-1*α*, MIP-1*β*, PDGF-BB, G-CSF, bFGF, RANTES, VEGF, GM-CSF, and TNF-*α*. The samples were analyzed using the Bio-Plex Luminex 200 System, and the results were calculated using Bio-Plex Manager 6.1 software (Bio-Rad Laboratories).

### 2.7. Statistical Analysis

All statistical analysis was performed using Prism 6 GraphPad software. Mann-Whitney test was used for the comparison of cytokine levels. P values of less than or equal to 0.05 were considered statistically significant.

## 3. Result

### 3.1. Establishment of Novel Protein IP Assay for Anti-EJ Autoantibody Detection

**Figure 1:**
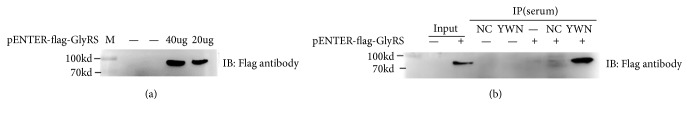
Establishment of unlabeled protein immunoprecipitation assay using HEK293 lysate overexpressing GlyRS with flag tag for detecting EJ autoantibody. (a) Overexpression of GlyRS in HEK293 cells by transient transfection of pENTER-flag- GlyRS plasmid was confirmed by immunoblot using flag antibody. (b) IP using HEK293 lysate overexpressing GlyRS with flag tag and sera from reference positive serum or healthy control serum.

We established a novel protein IP assay to detect anti-EJ autoantibody in IIM patients. The plasmid pENTER-flag- GlyRS with Flag tag was transiently transfected into HEK293 cells and the overexpression of flag-tagged GlyRS was detected at around 75 kDa by flag antibody (Figure 1(a)). As shown in Figure 1(b), reference serum (YWN) positive for anti-EJ autoantibodies specifically immunoprecipitated flag-tagged GlyRS overexpressed in HEK293 cells, but the health control serum did not.

### 3.2. Determination of Anti-EJ Autoantibody Prevalence in Chinese Myositis Cohort

**Figure 2:**
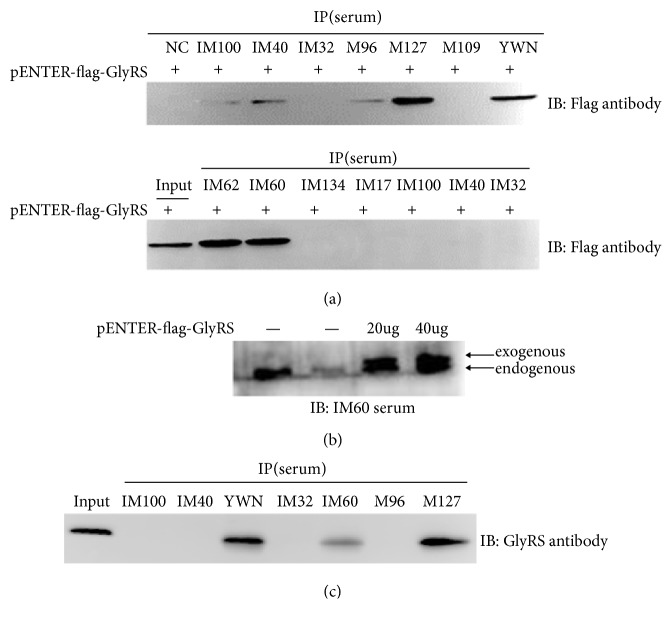
Detection of anti-EJ antibodies in IIM patients using unlabeled protein immunoprecipitation assay. (a) IP using HEK293 lysate overexpressing GlyRS with flag tag. Sera from 236 IIM patients and 20 normal controls were assayed by IP. All 4 anti-EJ antibody-positive sera (YWN, M127, IM60, IM62) are shown, as well as some representative anti-EJ negative IIM sera. (b) Immunoblotting of HEK293 cells extracts without (lanes 1 and 2) or with pENTER-flag-GlyRS plasmids transfection (lanes 3 and 4) using IM60 sera as primary antibody. Both exogenous and endogenous GlyRS in HEK293 cells were specifically identified by IM60 sera. (c) IP assay of K-562 cell lysates using patient sera and immunoblot of precipitated proteins using anti-GlyRS antibody.

To determine anti-EJ autoantibody prevalence, we performed the established protein IP assay in sera from 236 myositis patients using HEK293 cells extracts overexpressing flag-tagged GlyRS. Anti-EJ autoantibody was detected in 4 (YWN, M127, IM60, and IM62) of 236 patients with myositis, whereas the 20 normal control sera were negative (Figure 2(a)). Although sera from IM100, IM40, and M96 were immunoprecipitated with a little amount of flag-tagged GlyRS during the first screen, we could not detect the immunoprecipitated band anymore after we repeated again. To exclude the possibility that the autoantibody in sera of YWN, M127, IM60, and IM62 to any component of protein complex interacting with GlyRS protein would be theoretically immunoprecipitated with the flag-tagged GlyRS in HEK293 and thus appear as anti-EJ positive in screening [18], we also performed the immunoblotting of HEK293 cells extracts overexpressing flag-tagged GlyRS using four anti-EJ positive sera as primary antibody. As shown in Figure 2(b), both exogenous and endogenous GlyRS in HEK293 cells were identified from IM60 sera, and HEK293 cells extracts overexpressing flag-tagged GlyRS showed stronger band. YWN, M127, and IM62 had the same results as IM60 (data not shown). The conventional immunoprecipitation assay from K-562 cell lysates was performed using positive or negative patient sera and the precipitated proteins were confirmed by anti-GlyRS antibody (Figure 2(c)). The prevalence of anti-EJ autoantibody in our cohorts is about 1.7%.

### 3.3. Coexistence of Other Autoantibodies in Patients with Anti-EJ Antibody

Coexistence of anti-EJ antibody and other autoimmune diseases related antibodies was examined. Antibodies against dsDNA, nRNP/Sm, Sm, SSA, SSB, Scl-70, nucleosome, histone, and ribosomal P-protein were absent in all anti-EJ antibody-positive patients. In contrast, anti-Ro52 autoantibodies were found in all those patients with anti-EJ antibody (100%) and ANA were positive in three anti-EJ positive patients (Table 1). These results were consistent with previous findings that anti-Ro52 autoantibodies occur in 56-72% of anti-Jo-1 positive patients [19]. It is notable that anti-Jo-1 and anticentromere antibodies also coexist with anti-EJ in IM62 patient. This is the first myositis case with coexistence of anti-Jo-1 and anti-EJ antibodies, although simultaneous presence of anti-Jo-1 and anti-OJ has been previously described [20].

### 3.4. Clinical and Laboratory Data for Anti-EJ-Positive Patients

The clinical and laboratory data of anti-EJ-positive patients are summarized in Table 1. All patients were complicated with ILD as a common clinical feature. Three of them had clinical symptoms of pulmonary involvement such dry cough or dyspnea at initial visit, and patient YWN only presented remittent fever at disease onset and had ILD indicated by HRCT. The clinical manifestations and HRCT results of ILD were improved by methylprednisolone and cyclophosphamide therapy and their ILD did not have a fatal outcome. Muscle weakness accompanied with high CK (more than 1300 U/L) is another common feature among four anti-EJ-positive patients, although two of them did not complain muscle weakness at disease onset. Raynaud's phenomenon, Mechanic's hands, and rash only occurred in patients M127. IM62 coexisting with anti-JO 1, anti-centromere, and anti-EJ did not show any special phenotype.

### 3.5. Decline of Anti-EJ Levels in Two Patients during Disease Remission

**Figure 3:**
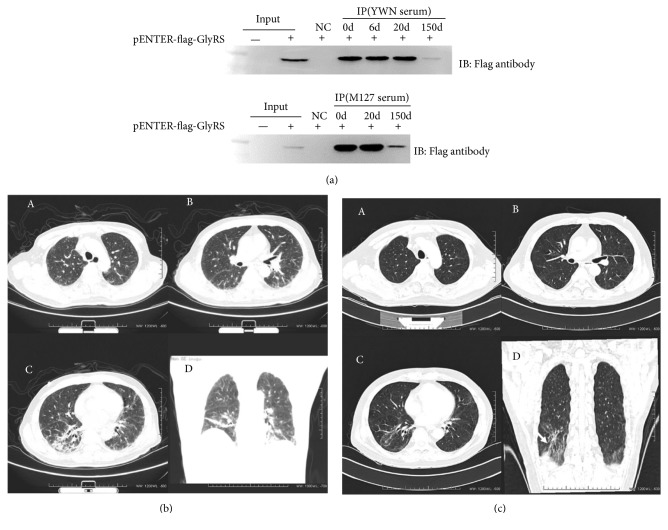
Decline of anti-EJ levels in two patients during disease remission. (a) The anti-EJ antibody levels were detected using the established protein IP assay in four longitudinal sera from patient YWN at 0d, 6d, 20d, 150d after treatment and three longitudinal sera from patient M127 at 0d, 20d, 150d after treatment. (b) Images from HRCT of the Chest on Admission. Selected axial CT images from the upper (Panel A), middle (Panel B), and lower (Panel C) thorax show bilateral reticular opacity and area of confluence along the bronchovascular bundle, localized traction bronchiectasis (arrows), as well as diffused ground-glass opacity, with most severe involvement of both lower lobes (Panel D). (c) Follow-up HRCT images obtained 5 Months after Admission. Selected axial CT images from the upper (Panel A), middle (Panel B), and lower (Panel C) thorax at the same levels as in Figure 1 show a decrease in bilateral reticular opacities and ground-glass. A coronal reformation (Panel D) shows mild traction bronchiolectasis (arrow). These findings are consistent with improvement of interstitial lung disease.

To assess the evolution of anti-EJ levels in anti-EJ positive patients, four longitudinal sera were retaken from patient YWN at 0 days, 6 days, 20 days, and 5 months after treatment and three longitudinal sera were collected from patient M127 at 0 days, 20 days, and 5 months after treatment. The anti-EJ antibody levels were detected using protein IP assay and the data showed that anti-EJ antibody levels were dramatically decreased in two patients at 5 months after treatment (Figure 3(a)). These two patients returned with obviously improved muscle weakness and persistent normal muscle enzymes level at 5 months after treatment. Their HRCT also showed significant improvement in interstitial pneumonia (Figures 3(b) and 3(c)). HRCT of patient YWN revealed a NSIP (nonspecific interstitial pneumonia) pattern characterized by bilateral reticular opacity and area of confluence along the bronchovascular bundle, localized traction bronchiectasis, and diffused ground-glass opacity especially among both lower lobes at initial visit (Figure 3(b)). After treated with methylprednisolone at the dosage of 40 mg/day, with tapering, and cyclophosphamide (0.6 g/2 weeks), HRCT showed significant improvement (Figure 3(c)).

### 3.6. Quantification of 27 Cytokines in Sera of Anti-EJ Antibody-Positive Patients

**Figure 4:**
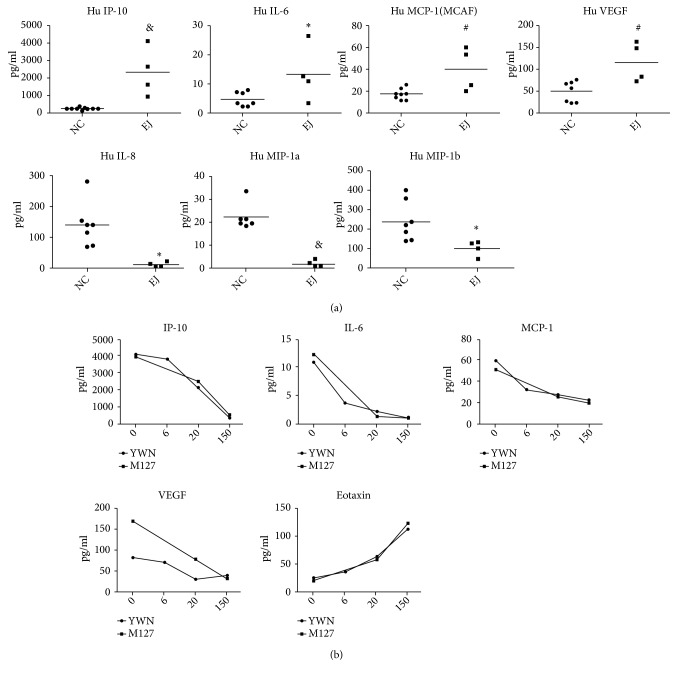
Quantification of 27 cytokines and chemokines in sera of 4 anti-EJ positive patients and 7 normal controls using bead-based multiplex assays. (a) The levels of seven cytokines showed significant difference in anti-EJ positive patients, among which IP-10, IL-6, MCP-1, and VEGF were significantly elevated in anti-EJ positive patients compared with NC (*∗*P < 0.05, ^#^P < 0.01, ^&^P < 0.001, vs. NC group). (b) The levels of IP-10, IL-6, MCP-1, and VEGF were gradually decreased in longitudinal sera from two patients and reached the levels of health control at 5 months after treatment, whereas eotaxin level was increased obviously after treatment.

Cytokines/chemokines are immunoregulatory mediators and play important roles in the pathogenesis of IIM. We performed the cytokines/chemokines profile in sera of anti-EJ patients using Bio-Plex Pro Human Cytokine 27-plex Assay. The data demonstrated that the serum levels of IP-10, IL-6, MCP-1, and VEGF are significantly elevated in patients with anti-EJ compared with the levels of health control, whereas the levels of IL-8, MIP-1*α*, and MIP-1*β* are significantly decreased, although to a lesser extent (Figure 4(a)). We also monitored cytokines/chemokines levels in longitudinal sera from patients YWN and M127 and found that the levels of IP-10, IL-6, MCP-1, and VEGF were gradually decreased and reached the levels of health control at 5 months after treatment, whereas eotaxin level was increased obviously after treatment (Figure 4(b)).

## 4. Discussion

Our report retrospectively screened anti-EJ antibody in sera from 236 Chinese patients with IIM by established immunoprecipitation assay and four patients were positive for anti-EJ, indicating that the prevalence of anti-EJ autoantibody in our IIM patient cohort is about 1.7%. The immunoprecipitation assay of flag-tagged GlyRS overexpressed in HEK293 cells was suitable to detect the decline of anti-EJ autoantibody level in longitudinal sera from two patients during disease remission, which is consistent with the improved muscle weakness and normal muscle enzymes level. We also confirmed the autoantibody in sera of anti-EJ positive patients, specifically targeting glycyl tRNA-synthetase, but not targeting any components of protein complex interacting with GlyRS protein by immunoblot of flag-tagged GlyRS using patient sera as primary antibody. Immunoprecipitation assay is suggestive to be more sensitive and specific than immunoblot since there is sometimes a moderate-to-poor agreement between immunoblot and IP [21]. Although radio-labeled immunoprecipitation assay is the gold standard technique to determine the presence of defined autoantibodies, it could not distinguish the autoantigens with similar molecular weight and the radioisotopes are used only in equipped laboratories under rigorous control. Moreover, radio-labeled immunoprecipitation assay also could not distinguish the primary autoantigens reacted with components of multiple complex [18]. Our unlabeled immunoprecipitation assay combined with immunoblot of overexpressing exogenous autoantigen can specifically detect the presence of defined autoantibodies. It is useful to monitor anti-EJ antibodies levels during disease process.

This report also aims to clarify the clinical features among ARS patients with anti-EJ autoantibody. Anti-EJ antibody was first identified in the serum of a single patient with Raynaud's phenomenon, weakness, possible mild sclerodactyly, and no evidence of myositis or ILD by* Targoff IN [22]. *After that, Anti-EJ antibodies were detected in the sera of five patients from 537 patients by immunoprecipitation of characteristic tRNAs and proteins. All 5 of the new patients had inflammatory myopathy, a typical DM rash, and ILD [23]. Recently, a multicenter study revealed that more than half of all patients with anti-EJ antibody were diagnosed with classical DM and clinically amyopathic DM (CADM) [8]. Patients with anti-EJ developed myositis later if they had ILD alone at the time of disease onset. In the present study, two out of four anti-EJ positive patients did not complain muscle weakness at disease onset but presented muscle weakness accompanied with high CK during the course of disease.* Hane H *developed an ELISA and immunoprecipitation method to detect 3 patients with myositis and 2 patients with other autoimmune diseases positive for anti-EJ. Two of 3 anti-EJ-positive patients with myositis were complicated with ILD and rash [24]. These results suggest that anti-EJ antibody is an important biomarker, especially in dermatologic clinics. Unlike the previously reported cases with anti-EJ, a typical DM rash was not common in our ASS patients with anti-EJ autoantibody, although they all presented ILD and more myositis patients.* Giannini M* also described three cases of anti-EJ-positive patients without skin involvement [25]. Our data and others suggested that anti-EJ-positive patients have distinct phenotypic features in different cohorts.

Many works have been done to unveil a group of cytokines known as type I interferon-regulated chemokines that plays a significant role in the development of IIM. With regard to cytokines, it has been demonstrated that CXCL9 and IP-10 are associated with anti-JO-1 antibody-associated ILD [26].* Bilgic H* and* Gono T* individually reported that the serum levels of IL-6 were significantly correlated with dermatomyositis disease activity [27, 28]. Consistent with previous studies, our data also demonstrated that the levels of IP-10, IL-6, MCP-1, and VEGF were significantly elevated in our patients with anti-EJ and were gradually decreased during disease remission of two patients. It is notable that the serum levels of IL-8 were obviously decreased in four patients positive with anti-EJ in the present study, but to a lesser extent.* Gono T *showed that IL-8 levels were significantly higher in the ILD subset of myositis especially in anti-MDA5-ILD, indicating that serum IL-8 is a useful predictor for fatal outcome due to ILD [28]. In the study of* Bilgic H*, the serum levels of IL-8 were also significantly lower in adult DM and juvenile DM than those in health controls [27]. The controversial data about serum IL-8 level of myositis patients may attribute to small number of patients, determination of myositis specific antibodies, and serum sampling time and so on.

There are several limitations to this study. First, there are smaller number of anti-EJ positive patients in the present study. Since the unlabeled immunoprecipitation assay is available, more anti-EJ ASS patients will be enrolled into our future study. Secondly, this study was retrospectively conducted. The levels of autoantibody and cytokine were only monitored in longitudinal sera from two patients. Future studies using longitudinal sera from more IIM patients are needed to determine whether it is useful to measure autoantibody titers and cytokine levels for disease activity, treatment response, and prognosis in distinct subsets of IIM.

## Figures and Tables

**Table 1:** Clinical features of anti-EJ antibody-positive patients in our cohort.

Patient ID	Symptom at initial visit	Diagnosis	ILD	fever	Muscle weakness	Gottron's sign	Heliotrope	Palmar erythema	Mechanics hands	arthritis	RP	High CK	Coexistence of other Ab
M127 F, 59Y	Rash, Weakness Dyspnea	DM	Y	N	Y	Y	N	Y	Y	Y	Y	Y	ANA, 1:160 Ro-52
IM60 F, 57Y	Dyspnea Fever Weakness	PM	Y	Y	Y	N	N	N	N	N	N	Y	Ro-52
IM62 F, 57Y	Dyspnea Arthralgia	PM	Y	N	Y	N	N	N	N	Y	N	Y	ANA, 1:80 Jo-1, Ro-52, anticentromere
Patient YWN M, 60Y	Fever	PM	Y	Y	Y	N	N	N	N	Y	N	Y	ANA, 1:80 Ro-52,

## Data Availability

The data used to support the findings of this study are available from the corresponding author upon request.

## References

[1] Mahler M., Miller F. W., Fritzler M. J. (2014). Idiopathic inflammatory myopathies and the anti-synthetase syndrome: a comprehensive review. *Autoimmunity Reviews*.

[2] Yao P., Fox P. L. (2013). Aminoacyl-tRNA synthetases in medicine and disease. *EMBO Molecular Medicine*.

[3] Hirakata M. (2005). Autoantibodies to aminoacyl-tRNA synthetases. *Internal Medicine*.

[4] Lazarou I. N., Guerne P.-A. (2013). Classification, diagnosis, and management of idiopathic inflammatory myopathies. *The Journal of Rheumatology*.

[5] Cavagna L., Castañeda S., Sciré C., Gonzalez-Gay M. A. (2018). Antisynthetase syndrome or what else? different perspectives indicate the need for new classification criteria. *Annals of the Rheumatic Diseases*.

[6] Gonzalez-Gay MA., Montecucco C., Selva-O'Callaghan A., Trallero-Araguas E., Molberg O., Andersson H. (2018). Timing of onset affects arthritis presentation pattern in antisyntethase syndrome. *Clinical and Experimental Rheumatology*.

[7] Connors G. R., Christopher-Stine L., Oddis C. V., Danoff S. K. (2010). Interstitial lung disease associated with the idiopathic inflammatory myopathies: what progress has been made in the past 35 years?. *Chest*.

[8] Hamaguchi Y., Fujimoto M., Matsushita T. (2013). Common and distinct clinical features in adult patients with anti-aminoacyl-trna synthetase antibodies: heterogeneity within the syndrome. *PLoS ONE*.

[9] Shi J., Li S., Yang H. (2017). Clinical profiles and prognosis of patients with distinct antisynthetase autoantibodies. *The Journal of Rheumatology*.

[10] Noguchi E., Uruha A., Suzuki S. (2017). Skeletal muscle involvement in antisynthetase syndrome. *JAMA Neurology*.

[11] Hervier B., Wallaert B., Hachulla E. (2010). Clinical manifestations of anti-synthetase syndrome positive for anti-alanyl-tRNA synthetase (anti-PL12) antibodies: a retrospective study of 17 cases. *Rheumatology*.

[12] Hervier B., Uzunhan Y., Hachulla E. (2011). Antisynthetase syndrome positive for anti-threonyl-tRNA synthetase (anti-PL7) antibodies. *European Respiratory Journal*.

[13] Hervier B., Devilliers H., Stanciu R. (2012). Hierarchical cluster and survival analyses of antisynthetase syndrome: phenotype and outcome are correlated with anti-tRNA synthetase antibody specificity. *Autoimmunity Reviews*.

[14] Lega J.-C., Fabien N., Reynaud Q. (2014). The clinical phenotype associated with myositis-specific and associated autoantibodies: a meta-analysis revisiting the so-called antisynthetase syndrome. *Autoimmunity Reviews*.

[15] Bohan A., Peter J. B. (1975). Polymyositis and dermatomyositis. *The New England Journal of Medicine*.

[16] Huang L., Wang L., Yang Y. (2018). Coexistence of anti-HMGCR and anti-MDA5 identified by an unlabeled immunoprecipitation assay in a chinese patient cohort with myositis. *Medicine*.

[17] Wang L., Huang L., Yang Y. (2018). Calcinosis and malignancy are rare in Chinese adult patients with myositis and nuclear matrix protein 2 antibodies identified by an unlabeled immunoprecipitation assay. *Clinical Rheumatology*.

[18] Targoff I. N., Trieu E. P., Miller F. W. (1993). Reaction of anti-OJ autoantibodies with components of the multi-enzyme complex of aminoacyl-tRNA synthetases in addition to isoleucyl-tRNA synthetase. *The Journal of Clinical Investigation*.

[19] Marie I., Hatron P. Y., Dominique S. (2012). Short-term and long-term outcome of anti-Jo1-positive patients with anti-Ro52 antibody. *Seminars in Arthritis and Rheumatism*.

[20] Gelpí C., Kanterewicz E., Gratacos J., Targoff I. N., Rodriguez-Sanchez J. L. (1996). Coexistence of two antisynthetases in a patient with the antisynthetase syndrome. *Arthritis & Rheumatology*.

[21] Cavazzana I., Fredi M., Ceribelli A. (2016). Testing for myositis specific autoantibodies: Comparison between line blot and immunoprecipitation assays in 57 myositis sera. *Journal of Immunological Methods*.

[22] Targoff I. N. (1990). Autoantibodies to aminoacyl-transfer RNA synthetases for isoleucine and glycine. two additional synthetases are antigenic in myositis. *The Journal of Immunology*.

[23] Targoff I. N., Trieu E. P., Plotz P. H., Miller F. W. (1992). Antibodies to glycyl–transfer rna synthetase in patients with myositis and interstitial lung disease. *Arthritis & Rheumatism*.

[24] Hane H., Muro Y., Watanabe K., Ogawa Y., Sugiura K., Akiyama M. (2014). Establishment of an ELISA to detect anti-glycyl-tRNA synthetase antibody (anti-EJ), a serological marker of dermatomyositis/polymyositis and interstitial lung disease. *Clinica Chimica Acta*.

[25] Giannini M., Notarnicola A., Dastmalchi M., Lundberg I. E., Lopalco G., Iannone F. (2016). Heterogeneous clinical spectrum of interstitial lung disease in patients with anti-EJ anti-synthetase syndrome: a case series. *Clinical Rheumatology*.

[26] Richards T. J., Eggebeen A., Gibson K. (2009). Characterization and peripheral blood biomarker assessment of anti-Jo-1 antibody-positive interstitial lung disease. *Arthritis & Rheumatism*.

[27] Bilgic H., Ytterberg S. R., Amin S. (2009). Interleukin-6 and type I interferon-regulated genes and chemokines mark disease activity in dermatomyositis. *Arthritis & Rheumatism*.

[28] Gono T., Kaneko H., Kawaguchi Y. (2014). Cytokine profiles in polymyositis and dermatomyositis complicated by rapidly progressive or chronic interstitial lung disease. *Rheumatology*.

